# Counteracting survival functions of EBNA3C in Epstein-Barr virus (EBV)-driven lymphoproliferative diseases by combination of SAHA and bortezomib

**DOI:** 10.18632/oncotarget.25341

**Published:** 2018-05-18

**Authors:** Kwai Fung Hui, Po Ling Yeung, Kam Pui Tam, Alan Kwok Shing Chiang

**Affiliations:** ^1^ Department of Pediatrics and Adolescent Medicine, Li Ka Shing Faculty of Medicine, The University of Hong Kong, Queen Mary Hospital, Pokfulam, Hong Kong SAR, China

**Keywords:** bortezomib, SAHA, Epstein-Barr virus, lymphoproliferative disease, EBNA3

## Abstract

Combination of suberoylanilide hydroxamic acid (SAHA) and bortezomib (SAHA/bortezomib) was shown to synergistically induce killing of lymphoblastoid cell lines (LCL) and Burkitt lymphoma (BL) of type III or Wp-restricted latency, both of which express EBNA3A, -3B and -3C proteins. We hypothesize that SAHA/bortezomib can counteract the survival functions conferred by the EBNA3 proteins. We tested the effect of SAHA/bortezomib on the survival of BL cell lines containing EBNA3A, -3B or -3C knockout EBV with or without the respective revertant EBNA3 genes. Isobologram analysis showed that SAHA/bortezomib induced significantly greater synergistic killing of EBNA3C-revertant cells when compared with EBNA3C-knockout cells. Such differential response was not observed in either EBNA3A or -3B revertant versus their knockout pairs. Interestingly, EBNA3C-knockout cells showed significant G2/M arrest whilst EBNA3C-revertant cells and LCLs escaped G2/M arrest induced by SAHA/bortezomib and became more susceptible to the induction of apoptosis. In parallel, SAHA/bortezomib induced stronger expression of p21^WAF1^ but weaker expression of p-cdc25c, an M-phase inducer phosphatase, in EBNA3C-expressing cells when compared with EBNA3C-knockout cells. SAHA/bortezomib also induced greater growth suppression of EBNA3C-expressing xenografts (EBNA3C-revertant and LCL) than that of EBNA3C-knockout xenografts in SCID mice. In conclusion, our data showed that SAHA/bortezomib could synergistically induce killing of BL and LCL through counteracting the survival functions of EBNA3C, providing a strong basis for clinical testing of this drug combination in patients with EBV-associated lymphoproliferative diseases.

## INTRODUCTION

Epstein-Barr virus (EBV) is a ubiquitous gamma herpesvirus that establishes life-long latent infections in normal human B cells in more than 90% of the world population. It usually causes asymptomatic infection in childhood but can also drive the uncontrolled proliferation of B cells through a concerted action of EBV latent proteins, including the EBV nuclear antigens (EBNA-1, -2, -LP, -3A, -3B and -3C) and latent membrane proteins (LMP-1, -2A and -2B) [1]. These viral proteins contribute to the pathogenesis of various EBV-associated lymphoproliferative diseases (LPDs) (e.g. post-transplant lymphoproliferative disorder (PTLD), hemophagocytic lymphohistiocytosis and X-linked lymphoproliferative disorder) and several types of lymphoid and epithelial malignancies (e.g. Burkitt lymphoma (BL), gastric carcinoma and nasopharyngeal carcinoma) [2]. There is an increasing dependence on EBV among tumor cells from type I, Wp-restricted, to type III latency which directly correlates with the number of viral genes expressed in the tumor cells [3].

Endemic BL, which expresses type I latency, is mainly found in children in Africa but a proportion of endemic BL (~15%) displays the Wp-restricted latency, in which the transcription of EBV latent genes is initiated in the W-promoter rather than the Q-promoter [2]. Frontline treatment regimens for PTLD and BL involve incorporation of chemotherapy agents and/or an anti-CD20 monoclonal antibody, rituximab. Intensive chemotherapy regimen can be associated with high toxicity in these patients whereas low-dose chemotherapy regimen can result in a high relapse rate [4]. Rituximab is found to be an effective drug in most cases but it profoundly suppresses normal B cell production for a period of at least 6 months and may be associated with potentially serious complications [5]. EBV resides in every tumor cell in the EBV-positive BL and PTLD patients. Infusion of EBV-specific cytotoxic T lymphocytes (CTLs) as a virus-targeted therapy is found to be effective for PTLD but the generation of EBV-specific CTLs of clinical grade requires a minimal duration of 4–6 weeks and can only be performed at a few specialized centers around the world [6]. Development of an alternative strategy to disrupt the survival mechanisms conferred by EBV may provide an attractive option for the treatment of Wp-restricted BL and PTLD as well as other types of EBV-driven lymphoproliferative diseases.

EBNA-LP, -3A, -3B and -3C are expressed in Wp-restricted BL and PTLD. EBNA-3A, -3B and -3C proteins share a homology domain of around 220 amino acids at the N-terminus in which around 20–30% identity between the EBNA3 proteins can be found. EBNA-3A and EBNA-3C (but not EBNA-3B) are essential for the growth transformation and maintenance of proliferation of LCLs [2, 7]. EBNA-3B was paradoxically demonstrated to have tumour suppressive function in BL cells [8]. Notably, EBNA-3C provided essential survival functions in the Wp-restricted BL cells and LCLs [7, 9, 10]. EBNA3C could control the phosphorylation of cdc25c and DNA damage response (DDR) pathway to disrupt G2/M cell cycle checkpoint for the continuous proliferation of LCLs [7, 10, 11]. The genes involved in apoptosis such as Bim, c-Myc, p53 and p21^WAF1^ were significantly down-regulated by EBNA-3C through epigenetic modification [9, 12, 13]. EBNA-3C could directly bind to epigenetic modifiers such as p300, histone deacetylase (HDAC)-1 and HDAC-2 [14, 15]. Besides affecting HDAC enzymes, EBNA-3C could also utilize the proteasomal degradation pathway to down-regulate the expression of various tumor suppressor genes, such as p21^WAF1^, and promote B cell proliferation [13]. We postulate that it is feasible to target the histone deacetylation and proteasomal degradation pathways for the treatment of EBV-driven lymphoproliferative diseases in which EBNA3C is expressed.

HDAC inhibitors could inhibit various types of HDAC enzymes and mediate potent anti-cancer effect in a wide range of malignancies [16]. We reported that FDA-approved HDAC inhibitors, including suberoylanilide hydroxamic acid (SAHA) and romidepsin, could induce growth arrest and apoptosis of EBV-positive gastric carcinoma and nasopharyngeal carcinoma cells by disrupting EBV latency [17–19]. HDAC inhibitors could also induce expression of p21^WAF1^ which was down-regulated by EBNA3C [13, 20]. Proteasome inhibitor, such as bortezomib, belongs to an emerging class of anti-cancer drugs which induce endoplasmic reticulum (ER) stress-related cell death through inhibition of the proteasomal degradation of unfolded proteins [21]. We and others had reported that combination of HDAC and proteasome inhibitors could mediate strong synergistic killing of cancer cells through generation of reactive oxygen species (ROS), activation of ER stress and induction of autophagy [21–25]. In addition, we found that combination of SAHA and bortezomib (SAHA/bortezomib) could preferentially induce killing of LCLs and BL cells of Wp-restricted latency, both of which express EBNA3A, -3B and -3C proteins [26]. These data suggested the involvement of the EBNA3 protein(s) in the cell death mechanism mediated by SAHA/bortezomib. Combination of HDAC and proteasome inhibitors was known to induce DNA damage response (DDR) in various tumor cells [27, 28]. In response to DDR, cells were arrested at cell cycle checkpoints in order to provide sufficient time for the cells to repair the damaged DNA [29]. Ataxia telangiectasia mutated/Rad3-related (ATM/ATR) pathways were known to mediate G1 arrest through p53/p21 pathway and G2/M arrest through inactivation of cdc25c [30, 31]. EBNA-3C was shown to release the DDR-induced G2/M arrest through dysregulated cdc25c phosphorylation when cells were exposed to nocodazole [11]. Yet, the effects of combination of HDAC and proteasome inhibitors on the cell cycle progression and survival of EBNA-3 expressing cells have not been investigated.

We hypothesize that SAHA/bortezomib can induce synergistic killing of BL and LCLs through targeting the survival functions of EBNA3 proteins. To test this hypothesis, we examined the effect of SAHA/bortezomib on the survival of BL cell lines which harbor EBNA3A or -3B or -3C knockout EBV with or without the individual revertant. We found that EBNA3C played a more important role in the synergistic killing of BL cells and LCLs when compared with EBNA3A and EBNA3B. Our data suggested that SAHA/bortezomib targeted the survival functions of EBNA3C protein in BL and LCLs. This is the first study to show that combination of HDAC/proteasome inhibitors can indeed target latent viral protein function in EBV-associated LPDs.

## RESULTS

### Combination of HDAC and proteasome inhibitors (i.e. SAHA /bortezomib) synergistically inhibited the proliferation of EBNA3C-expressing BL cells

**Figure 1:**
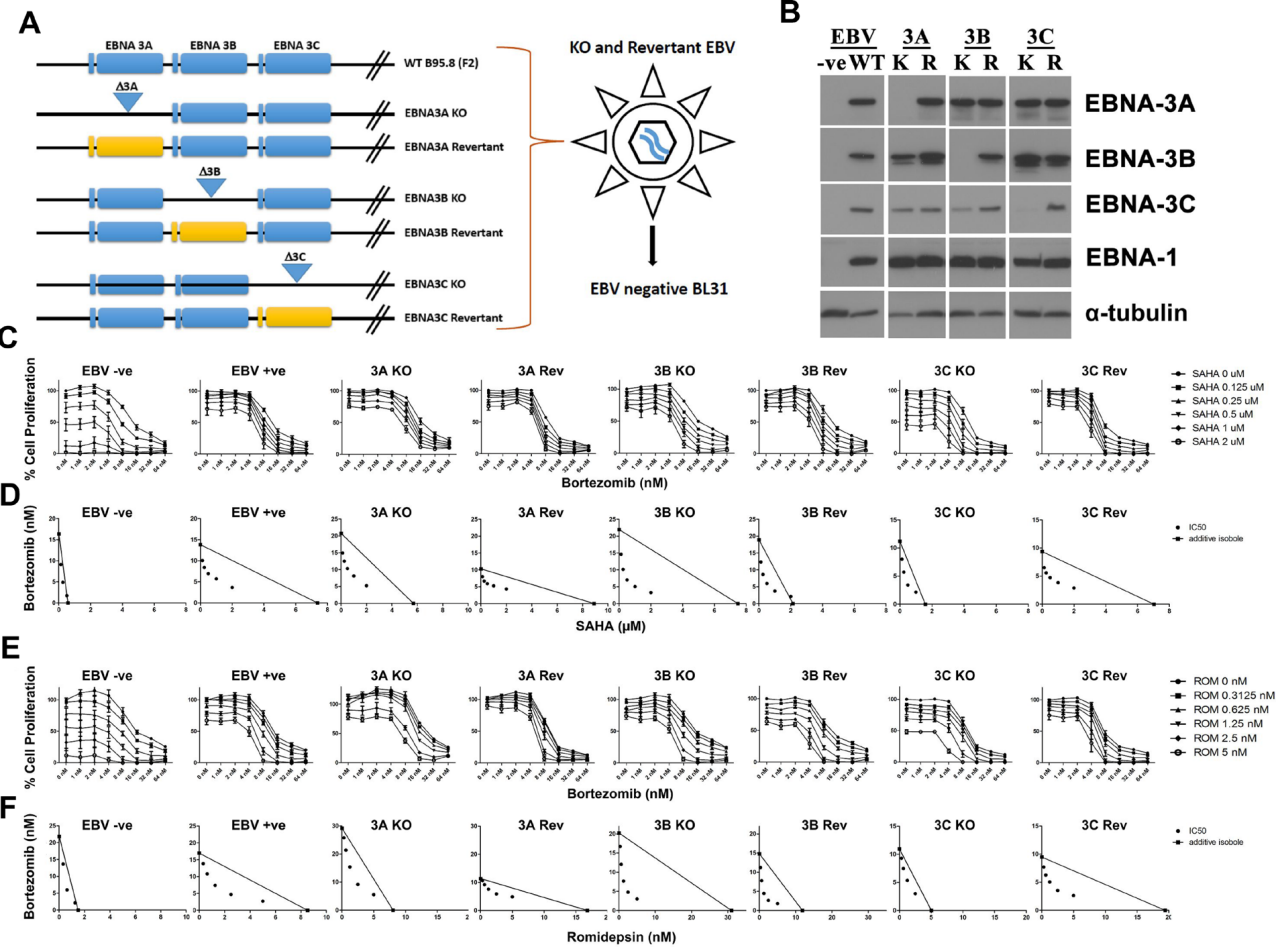
Effects of combination of HDAC and proteasome inhibitors on cell proliferation of EBNA3C-knockout (3C-KO) and EBNA3C-expressing (3C-Rev) BL 31 cells (**A**) Schematic diagram illustrating the generation of EBV-negative, WT, EBNA3A/-3B/-3C-knockout (KO) and -revertant (Rev) BL 31cells. (**B**) Western blot analysis showing the expression of EBNA3A/-3B/-3C and EBNA1 proteins in EBV-negative, -WT, EBNA3A/3B/-3C-knockout (K) and -revertant (R) BL 31 cells. α-tubulin was detected as a loading control. (**C**–**F**) MTT analyses showing the combinatorial effect of SAHA/bortezomib on the proliferation of EBV-negative, -WT, EBNA3A/3B/-3C knockout and revertant BL 31 cells. (C) The BL31 cell lines were treated with combination of SAHA (0, 0.125, 0.25, 0.5, 1, 2 μM) and bortezomib (0, 1, 2, 4, 8, 16, 32, and 64 nM) for 24 hr. Percentages of proliferation of treated cells compared with untreated cells were determined. (D) Synergisms of proliferation inhibition of the two cell lines were analyzed by isobologram analysis. (E) The BL31 cell lines were treated with combination of romidepsin (0, 0.3125, 0.625, 1.25, 2.5, 5 nM) and bortezomib (0, 1, 2, 4, 8, 16, 32, and 64 nM) for 24 hr. Percentages of proliferation of treated cells compared with untreated cells were determined. (F) Synergisms of proliferation inhibition of the two cell lines were analyzed by isobologram analysis. Error bars represent the standard error of mean (SEM) of data obtained in at least three independent experiments.

We had reported that combination of HDAC and proteasome inhibitors could induce specific synergistic killing of EBV-positive BL cells and LCLs which express EBNA3 proteins. To investigate the essential role of EBNA-3 proteins in the survival of cells, we tested that effects of combination of HDAC and proteasome inhibitors on the proliferation of eight different BL31 cell lines, including a EBV-negative BL31 cell line (EBV -ve) and BL31 cell lines infected with wild type EBV (EBV +ve), EBNA-3A-knockout (3A-KO), EBNA-3B-knockout (3B-KO), EBNA-3C-knockout (3C-KO) EBV and their revertants (i.e. 3A-Rev, 3B-Rev and 3C-Rev). A schematic diagram showing the genomic structures of the individual EBNA3 genes knockout (KO) and revertant (Rev) BL31 cell lines is shown in Figure 1A [32]. The patterns of expression of EBV latent proteins of the eight BL31 cell lines were detected by western blot analysis. Specific knockouts of the respective EBNA3 genes were observed (Figure 1B).

The eight BL31 cell lines were treated with combination of SAHA and bortezomib for 24 hours. The treated cells were assayed for percentage of cell proliferation by MTT assay (Figure 1C). Dose-dependent cytotoxicity was observed in all BL31 cell lines treated with either drug alone. Of note, the EBNA3C-positive cell lines (i.e. EBV +ve, 3A-KO, 3A-Rev, 3B-KO, 3B-Rev and 3C-Rev cells) were more resistant to the killing by SAHA in contrast to the obvious dose-dependent killing by SAHA in EBV-ve and 3C-KO cells when bortezomib was not administrated. Combination indexes (CI) between 8 nM bortezomib and 1 μM SAHA on the killing of cell lines were calculated. The CI on EBV-ve, WT, 3A-KO, 3A-Rev, 3B-KO, 3B-Rev, 3C-KO and 3C-Rev were 0.601, 0.442, 0.675, 0.596, 0.32, 0.456, 0.484 and 0.317, respectively. The drug combination was synergistic in the killing of all these cell lines as the CI were all less than 1. The lower CI on 3C-Rev than that on 3C-KO cells indicated that SAHA/bortezomib induced a relatively stronger synergistic killing of the 3C-Rev cells than the 3C-KO cells. The synergism between SAHA/bortezomib was further analyzed by isobologram analysis (Figure 1D). The isoboles lie more to the right of the additive isoboles in the EBV +ve cells than the EBV -ve cells, indicating stronger synergisms of bortezomib and SAHA in their anti-proliferative effects on EBV-positive cells than those on EBV-negative cells. In addition, higher degree of synergism between SAHA/bortezomib was observed in 3C-Rev cells when compared with 3C-KO cells. Such differential response was not found in other EBNA-3 knockout and revertant pairs (i.e. 3A-KO vs 3A-Rev; 3B-KO vs 3B-Rev). Similar patterns of synergistic killing of BL cells (i.e. specific synergism in EBV +ve and 3C-Rev) could also be observed upon treatment with combination of bortezomib and romidepsin, another HDAC inhibitor (Figure 1E and 1F). These data suggest that combination of HDAC and proteasome inhibitors might target the survival pathways activated by EBNA3C in the EBV-positive BL cells.

### SAHA/bortezomib also induced synergistic killing of spontaneous LCLs established from pediatic PTLD patients

**Figure 2:**
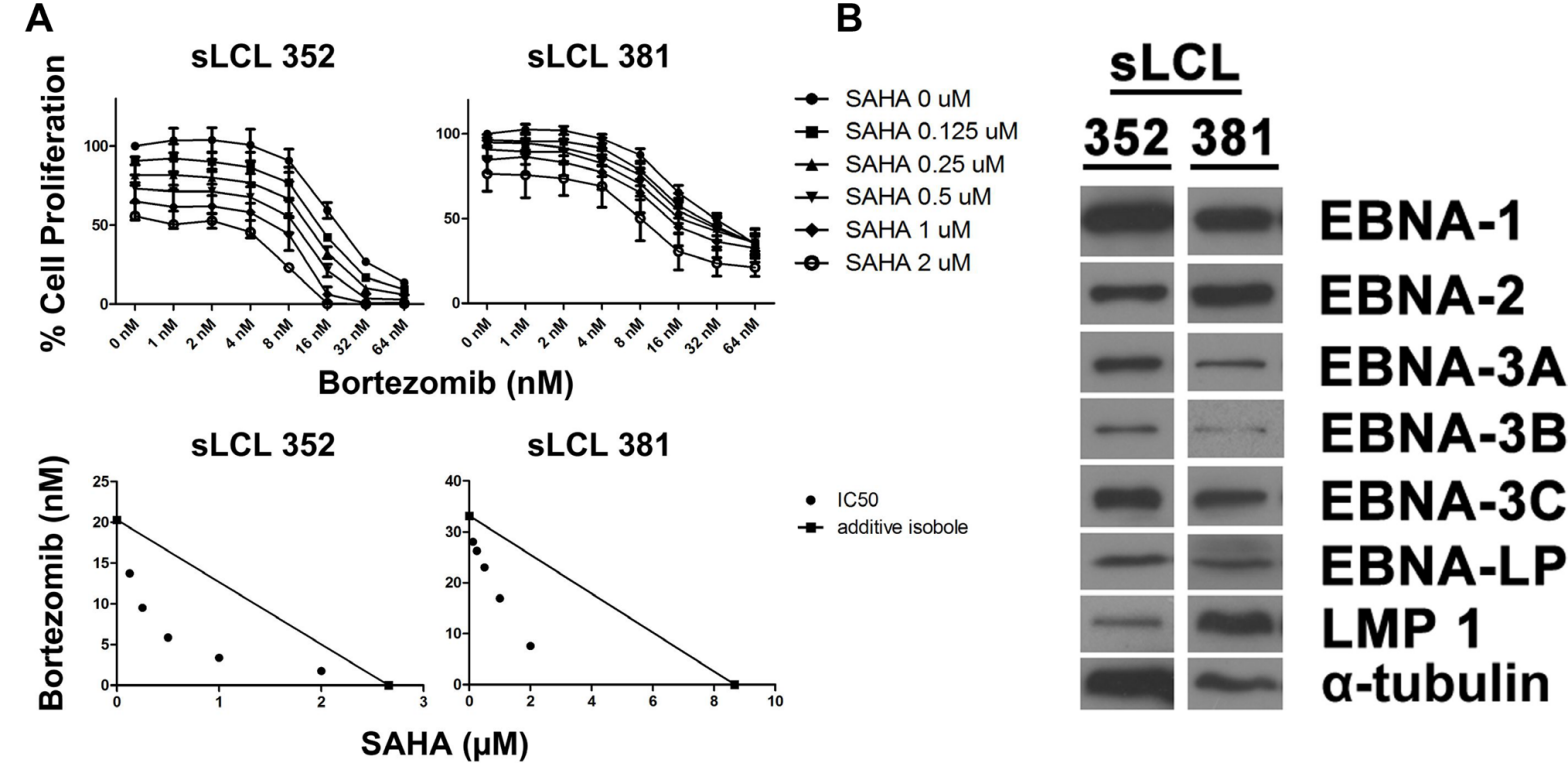
Effects of combination of SAHA and bortezomib on proliferation of lymphoblastoid cell lines (LCLs) Two spontaneous LCLs, sLCL 352 and sLCL 381, were treated with combination of SAHA (0, 0.125, 0.25, 0.5, 1, 2 μM) and bortezomib (0, 1, 2, 4, 8, 16, 32, and 64 nM) for 48 hr. (**A**) Percentages of cell proliferation were determined and isobologram analyses were performed. Error bars signify the standard error of mean (SEM) of data obtained in 3 independent experiments. (**B**) Western blot analysis showing the expression of EBNA1, -2, -3A, -3B, -3C, -LP and LMP1 in sLCL 352 and sLCL 381. α-tubulin was detected as a loading control.

Next, we investigated the effect of SAHA/bortezomib on the killing of spontaneous LCLs established from pediatic PTLD patients. Two spontaneous LCLs, sLCL 352 and sLCL 381, were treated with combination of SAHA and bortezomib for 48 hr. The relative cell proliferation was determined by MTT assay and the dose-response curves are shown in Figure 2A. While each drug was able to reduce the proliferation of the sLCLs in a dose-dependent manner, the combination of SAHA/bortezomib yielded much stronger anti-proliferative effect. In the isobolograms, the isoboles for IC50 lie below the additive isoboles for both sLCLs, suggesting synergism of SAHA/bortezomib in their anti-proliferative effects (Figure 2A). The expression of EBV latent proteins in sLCL 352 and sLCL 381 was detected by western blot analysis. The latent proteins of EBV, including EBNA1, -2, -3A, -3B, -3C, -LP and LMP1, were detected in both sLCL (Figure 2B). The results confirmed the latency III expression pattern of sLCL 352 and sLCL 381 and supported our postulation that SAHA/bortezomib could target the function of proteins in EBV latency III-expressing cell lines.

### The synergistic killing by SAHA/bortezomib was related to decreased G2/M arrest and increased apoptosis of EBNA3C -positive cells

**Figure 3:**
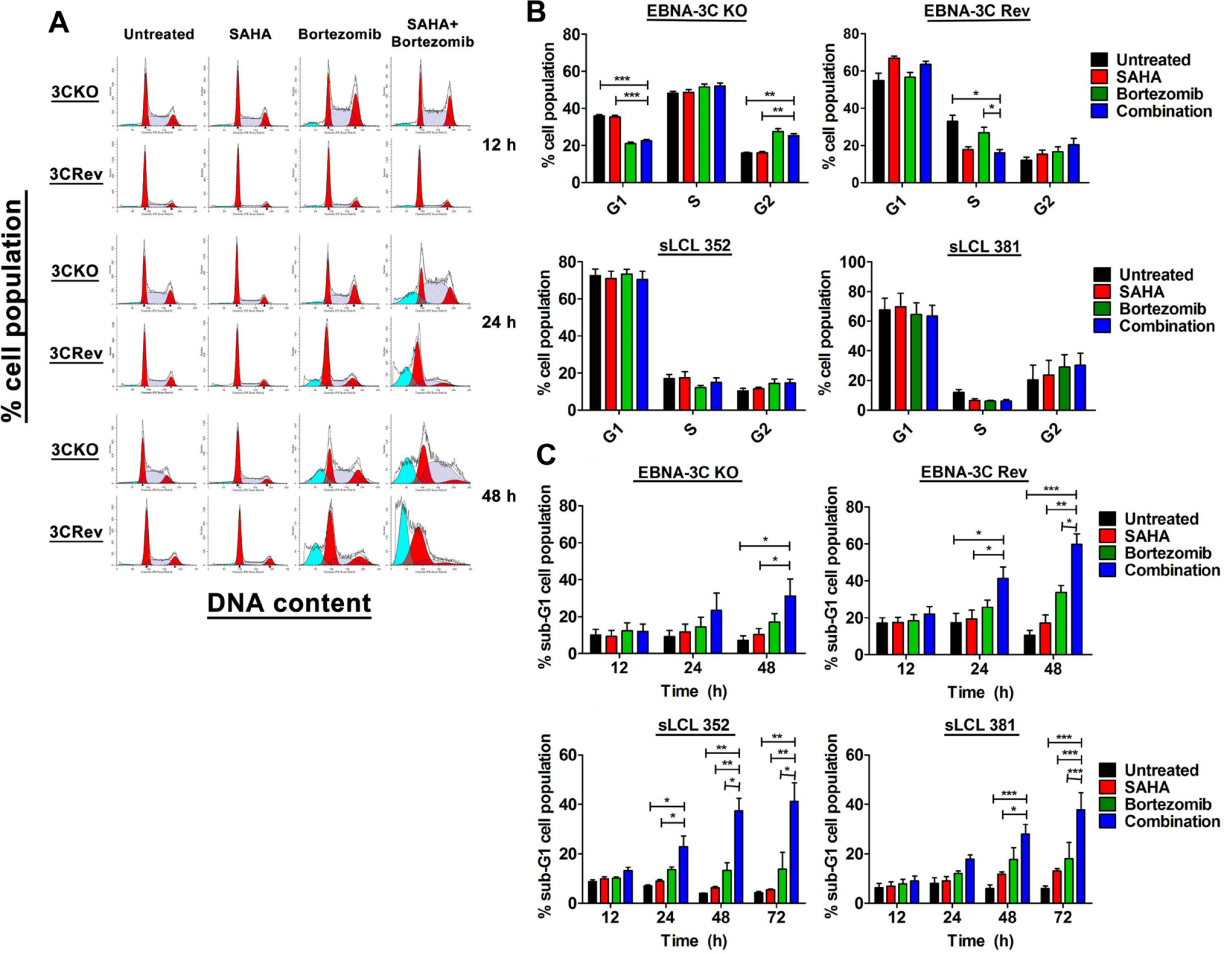
Effects of SAHA/bortezomib on the progression of cell cycle and induction of apoptosis of EBNA3C-knockout (KO) and -revertant (Rev) BL cells and spontaneous LCLs (sLCL) EBNA3C-KO, EBNA3C-Rev BL cells, sLCL 352 and sLCL 381, were treated with combination treatment of 1 μM SAHA and 8 nM bortezomib or either drug alone for 12, 24, 48 or 72 hr. The treated cells were stained with propidium iodide and subjected to analysis of cellular DNA content by flow cytometry. (**A**) A representative data set of cell cycle patterns of EBNA3C-KO and -Rev BL cells upon treatments was presented. (**B**) The percentages of cells in G1, S and G2/M phases at 12 hr were analyzed for statistical significance using One-way ANOVA Dunnett's Multiple Comparison Test. (**C**) The percentages of sub-G1 population were analyzed for statistical significance using One-way ANOVA Dunnett's Multiple Comparison Test. *p*-value < 0.05 was considered as statistically significant. (^*^p < 0.05, ^**^p < 0.01, ^***^p < 0.001 compared with SAHA/Bortezomib). Error bars signify the standard error of mean (SEM) of data obtained in 3 independent experiments.

To examine the effects of SAHA/bortezomib on the induction of cell cycle arrest in 3C-KO and 3C-Rev cell lines, cell cycle analyses were performed. Both cell lines were treated with either 1 μM SAHA, 8 nM Bortezomib or their combination for 12, 24 and 48 hr. The treated cells were collected and stained with propidium iodide and subjected to analysis of cellular DNA content by flow cytometry. The mean of percentages of G1, S, G2 and sub-G1 populations were calculated from 3 independent experiments. One representative set of cell cycle patterns of the 3C-KO and 3C-Rev BL31 cell lines upon treatments with SAHA/bortezomib, was presented in Figure 3A. At 12 hr post-treatment, bortezomib alone or combination of SAHA/bortezomib induced a significant G2/M phase arrest in the 3C-KO cells. However, such G2/M arrest was not observed in neither 3C-Rev BL cells, sLCL 352 and sLCL 381, suggesting that the expression of EBNA-3C could allow these cells to bypass the G2/M arrest induced by SAHA/bortezomib (Figure 3B). SAHA/bortezomib-induced G2/M arrest was also clearly observed in the EBV-ve BL31 cells but not in the WT EBV-infected BL31 cells (Supplementary Figure 1). Beside, relatively high percentage of cells in S phase was observed in both EBV-ve and 3C-KO BL31 cells but such population was lower in the EBV+ve and 3C-Rev BL31 cells (Figure 3B and Supplementary Figure 1). On the other hand, SAHA/bortezomib induced a greater percentage of sub-G1 populations in 3C-Rev cells when compared to that of 3C-KO cells (63% versus 35%) at 48 hr post-treatment, indicating induction of a stronger degree of apoptosis in 3C-Rev cells when compared with the 3C-KO cells. Taken together, our data implied that the expression of EBNA-3C could help the EBV-infected cells to override the G2/M arrest induced by SAHA/bortezomib, rendering the cells more sensitive to the induction of apoptosis by the drug combination.

### Increased synergistic killing and decreased G2/M arrest were observed in a second pair of BL cell lines (EBNA3C-KO and EBNA3C-Rev BL2 cells)

**Figure 4:**
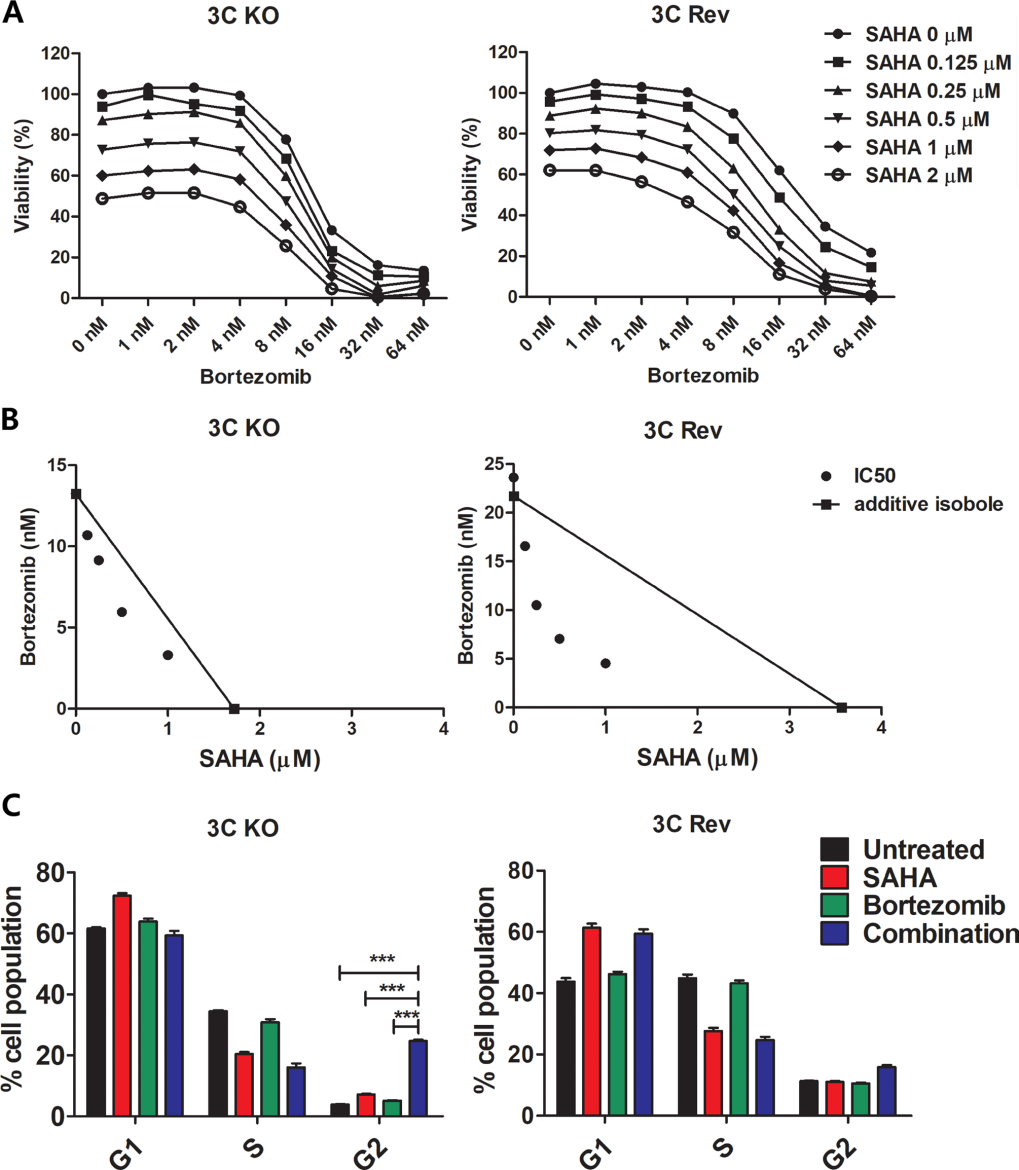
Effects of combination of SAHA and bortezomib on cell proliferation and cell cycle progression of EBNA3C-knockout and EBNA3C-expressing BL2 cells (**A**) MTT analyses showing the combinatorial effect of SAHA/bortezomib on the proliferation of 3C-KO and 3C-Rev BL2 cells. The cells were treated with combination of SAHA (0, 0.125, 0.25, 0.5, 1, 2 μM) and bortezomib (0, 1, 2, 4, 8, 16, 32, and 64 nM) for 24 hr. Percentages of proliferation of treated cells compared with untreated cells were determined. (**B**) Synergisms of proliferation inhibition of the two cell lines by SAHA/bortezomib were analyzed by isobologram analysis. (**C**) 3C-KO and 3C-Rev BL2 cells were treated with combination of 1 μM SAHA and 8 nM bortezomib or either drug alone for 12 hr. The treated cells were stained with propidium iodide and subjected to analysis of cellular DNA content by flow cytometry. The percentages of cells in G1, S and G2/M phases were analyzed for statistical significance using One-way ANOVA Dunnett's Multiple Comparison Test. Error bars represent the standard error of mean (SEM) of data obtained in at least three independent experiments.

Because the EBNA-3C KO and EBNA-3C Rev BL 31 cell lines were generated separately by infection, selection of subclones of the cell lines from these cell cultures might contribute to the changes in response to the treatment by SAHA/bortezomib. To eliminate this possibility, we tested the synergistic effects of SAHA/bortezomib on the killing of a second pair of BL cell lines (EBNA3C-KO and EBNA3C-Rev BL2 cells) [32]. The BL2 cells were treated with SAHA/bortezomib for 24 hours followed by determination of the percentage of cell proliferation by MTT assay. The synergism between SAHA and bortezomib was analyzed by isobologram analysis (Figure 4A and 4B). Consistent with the finding on the BL31 cells, higher degree of synergism between SAHA/bortezomib was observed in 3C-Rev BL2 cells when compared with 3C-KO BL2 cells. Interestingly, more significant G2/M arrest could also be observed in the 3C-KO BL2 cells when compared with the 3C-Rev BL2 cells (Figure 4C). Taken together, despite a difference in the genetic backgrounds between the BL31 and BL2 cell lines [32], the EBNA-3C mediated G2/M checkpoint dysregulation and synergistic cell death in response to SAHA/bortezomib could be consistently observed in both cell lines.

### SAHA/bortezomib induced stronger expression of p21^WAF1^ but weaker expression of p-cdc25c in EBNA3C-expressing cells when compared with EBNA3C-knockout cells

**Figure 5:**
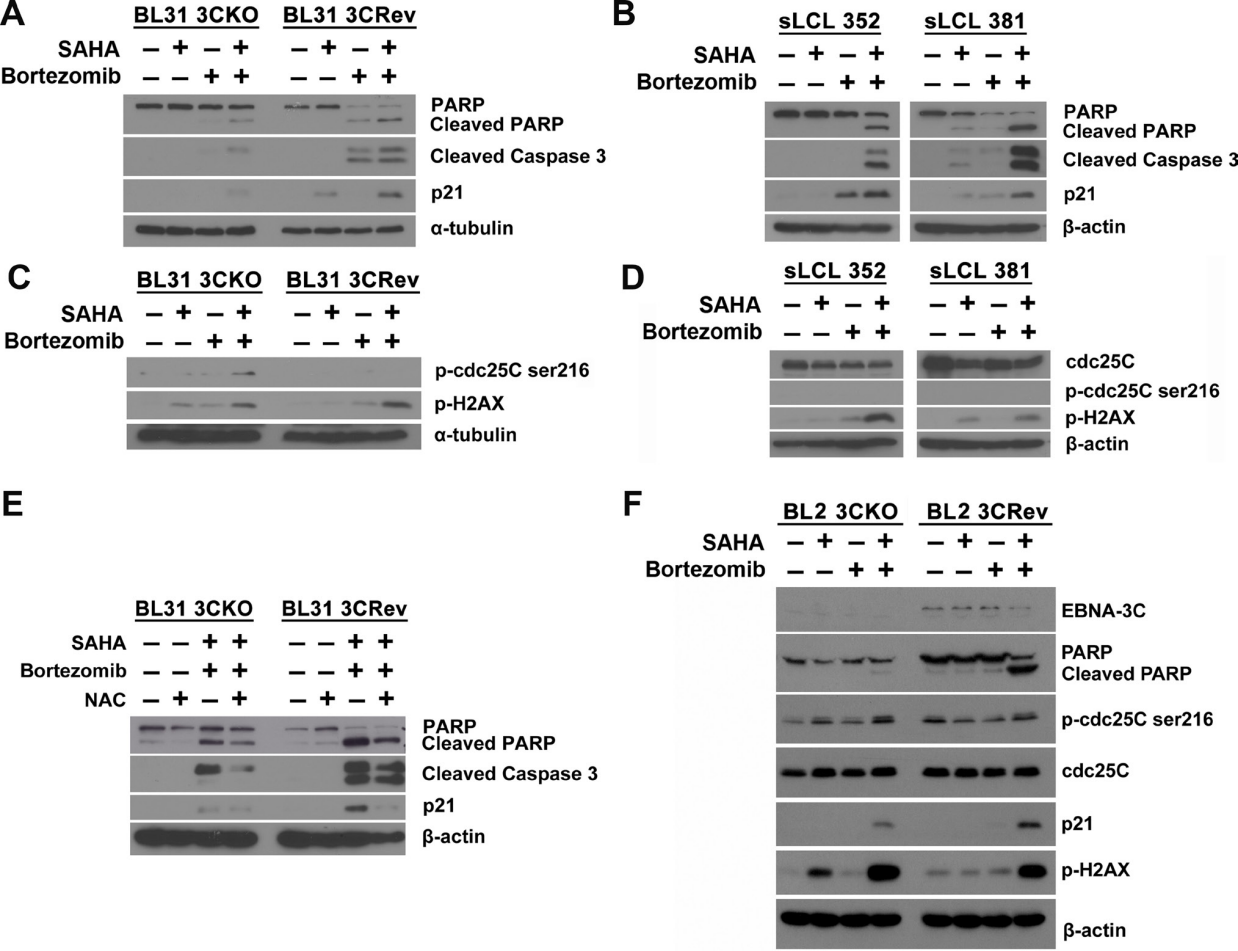
Effects of SAHA/bortezomib on the expression of cellular molecules related to EBNA3C signaling Western blot analysis showing the effect of SAHA/bortezomib on the expression of apoptotic markers in (**A**) EBNA3C-knockout (KO) and -revertant (Rev) BL 31 cells and (**B**) sLCL 352 and sLCL 381. EBNA3C-KO, EBNA3C-Rev, sLCL 352 and sLCL 381 were treated with combination of 1 μM SAHA and 8 nM bortezomib or either drug alone for 12 hr. Cell lysates were prepared and the expression of PARP, cleaved PARP, cleaved caspase-3 and p21^WAF1^ was examined by western blotting. α-tubulin or β-actin was detected as a loading control. Western blot analysis showing the expression of total cdc25c, p-cdc25c-ser216 and p-H2AX in (**C**) EBNA3C-KO cells, EBNA3C-Rev cells, (**D**) sLCL 352 and sLCL 381 upon treatment with SAHA/bortezomib. EBNA3C-KO cells, EBNA3C-Rev cells, sLCL 352 and sLCL 381 were treated with combination of 1 μM SAHA and 8 nM bortezomib or either drug alone for 12 hr. Protein samples were extracted and the expression of cdc25c, p-cdc25c-ser216 and p-H2AX was examined by western blotting. α-tubulin or β-actin was detected as a loading control. (**E**) Western blot analysis showing the potential role of reactive oxygen species (ROS) generation in the apoptosis of EBNA3C-KO and -Rev BL cells upon treatment with SAHA/bortezomib. EBNA3C-KO and -Rev BL cells were pretreated with 12 mM N-acetyl-cysteine (NAC) for 1 hr followed by combination treatment of 1 μM SAHA and 8 nM bortezomib for 12 hr. Protein samples were extracted and the expression of PARP, cleaved PARP, cleaved caspase-3 and p21^WAF1^ was analyzed by western blotting. β-actin was detected as a loading control. (**F**) Western blot analysis showing the expression of cellular molecules that are related to EBNA3C signaling in BL2 cells. EBNA3C-KO and EBNA3C-Rev BL2 cells were treated with combination of 1 μM SAHA and 8 nM bortezomib or either drug alone for 16 hr. Cell lysates were prepared and the expression of EBNA3C, PARP, cleaved PARP, cdc25c, p-cdc25c-ser216 and p-H2AX and p21^WAF1^ was examined by western blotting. β-actin was detected as a loading control.

**Figure 6:**
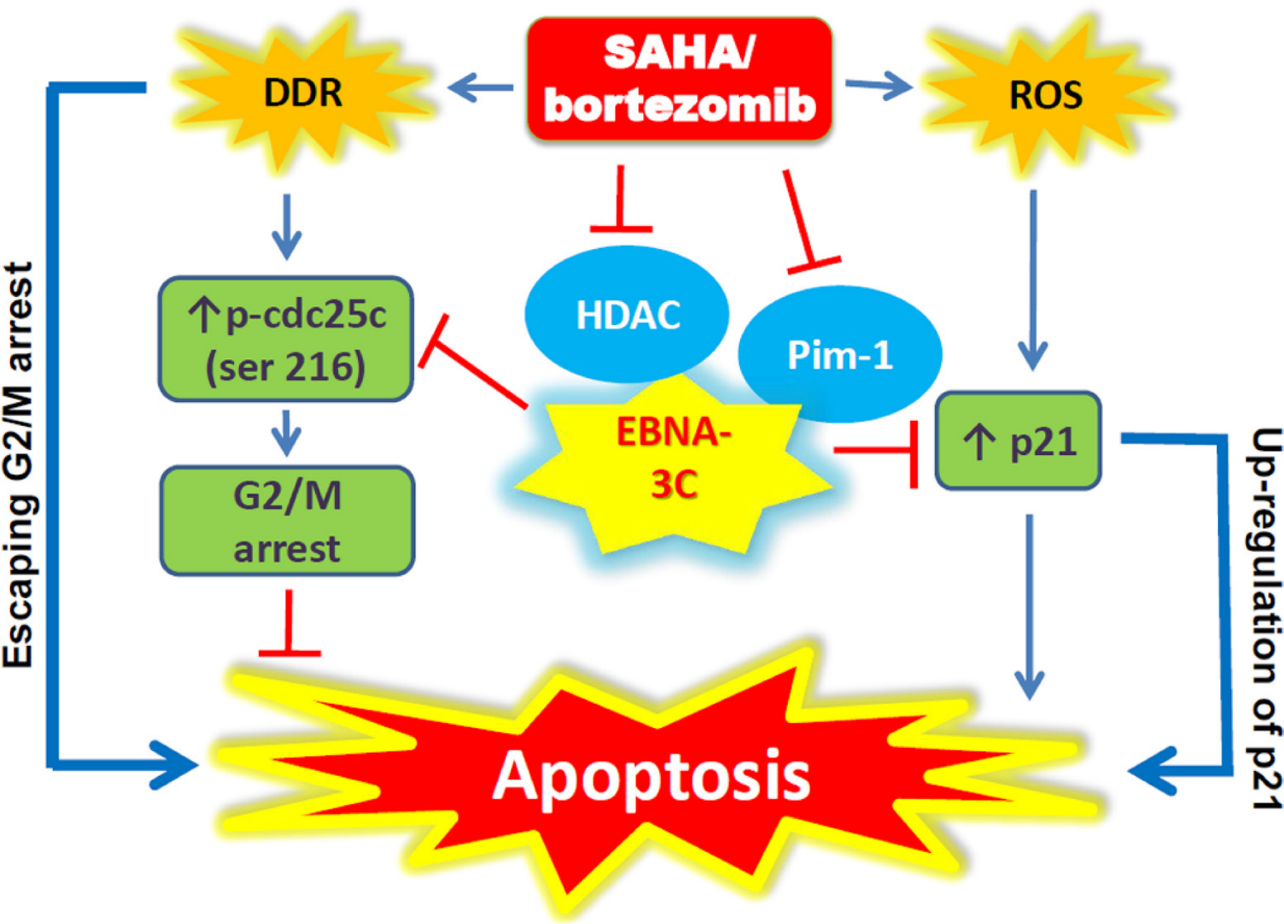
Schematic diagram illustrating the potential mode of action of SAHA/bortezomib in EBNA3C-expressing cells

We had reported that SAHA/bortezomib could up-regulate the expression of p21^WAF1^ (inducer of apoptosis) in EBNA3C-expressing cells [26]. In addition, EBNA-3C can release the DNA damage response (DDR)-induced G2/M arrest through dysregulated cdc25c phosphorylation [11]. 3C-KO, 3C-Rev BL cells, sLCL 352 and sLCL 381 were treated with combination of 1 μM SAHA and 8 nM bortezomib or either drug alone for 12 hr. Protein samples were extracted and the expression of p21^WAF1^, p-cdc25c and p-H2AX (a key marker of DDR) was examined by western blot analysis (Figure 5). When compared with either drug alone, SAHA/bortezomib induced a significantly stronger cleavage of PARP and caspase-3 along with stronger expression of p21^WAF1^ in the EBNA3C-expressing cells (i.e. 3C-Rev, sLCL352 and sLCL381)(Figure 5A and 5B). Up-regulation of p-H2AX proteins level by SAHA/bortezomib was observed in all four cell lines suggesting DDR was induced regardless of the presence of EBNA3C (Figure 5C and 5D). On the other hand, the expression of p-cdc25C (ser216), an upstream inducer of G2/M arrest, was only up-regulated in 3C-KO but not in 3C-Rev BL31 cells or sLCL upon the treatment with SAHA/bortezomib (Figure 5C–5E). Increased expression of p-cdc25C, p-H2AX and p21^WAF1^ could also be observed in the 3C-KO versus 3C-Rev BL2 cells in response to the treatment with SAHA/bortezomib (Figure 5F). These data suggested that the synergistic killing and dysregulation of G2/M arrest in the EBNA3C-expressing cells might be related to the induction of DDR, up-regulation of p21^WAF1^ and decreased phosphorylation of cdc25c (Figure 6).

### SAHA/bortezomib suppressed the growth of EBNA3C-expressing BL and LCL *in vivo*

**Figure 7:**
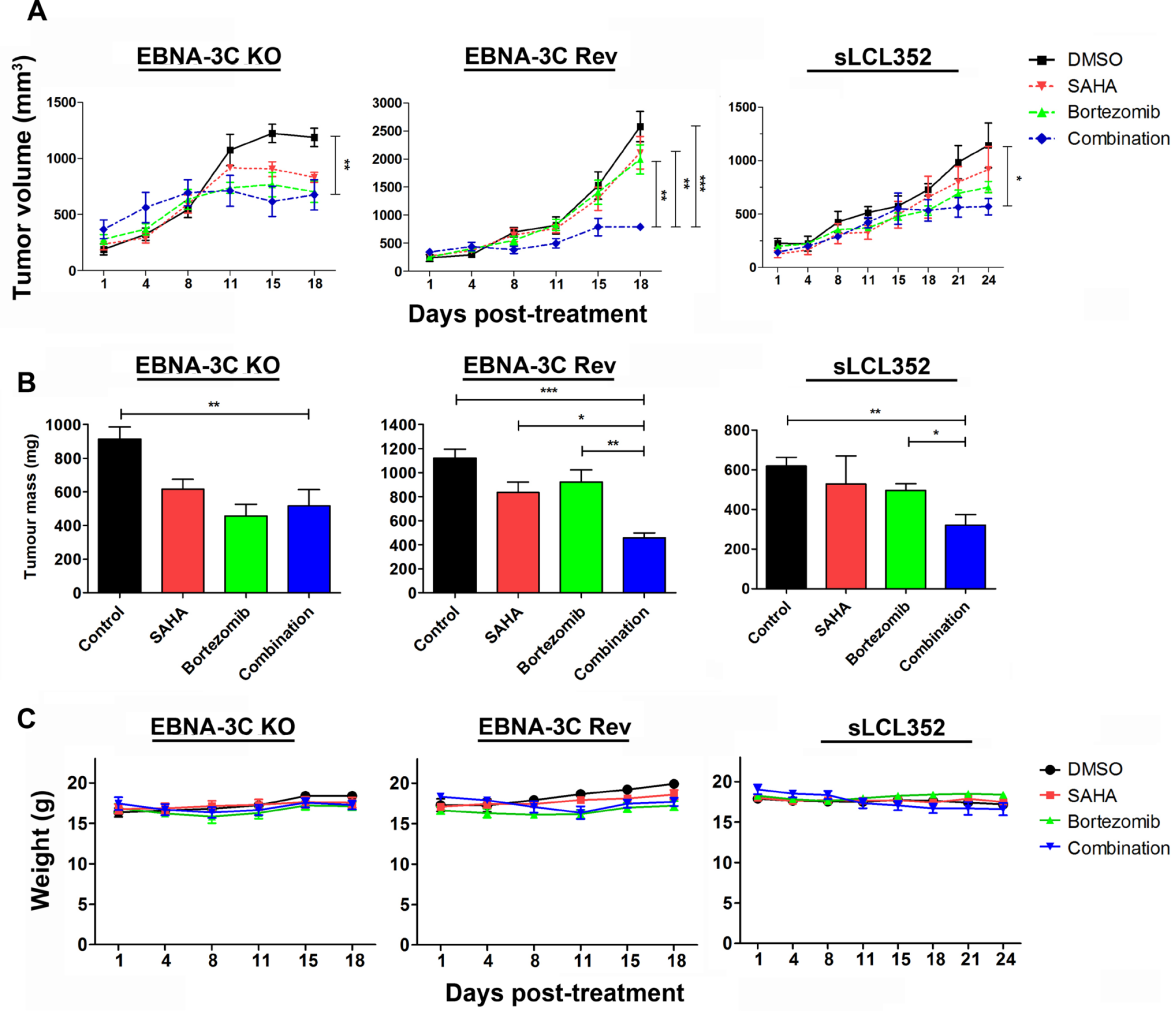
Effects of SAHA/bortezomib on the growth suppression of EBNA3C-knockout (KO), EBNA3C-revertant (Rev) and sLCL 352 xenografts in SCID mice EBNA3C-KO, EBNA3C-Rev and sLCL 352 (1 × 10^7^ cells) were subcutaneously injected into the right flanks of SCID mice. When the tumours were palpable, the mice were treated with combination of 50 mg/kg SAHA and 60 μg/kg bortezomib (*n* = 5) or either drug alone for 5 days per weak over 18 days (or 24 days for sLCL 352) by intraperitoneal injection. (**A**) The size of tumours during the period of experiment was measured twice weekly using a caliper. Data are presented as the mean tumour volumes of mice in both treatment and control groups on the days post-treatment (1, 4, 8, 11, 15, 18 days). The tumours were dissected out at the end of experiment (18 days post-treatment). (**B**) The average of tumour masses of mice of control and treated groups were shown. (**C**) The mice were weighed at 1, 4, 8, 11, 15 and 18 days post-treatment. The results were analyzed for statistical significance using One-way ANOVA Dunnett's Multiple Comparison Test. *P* value less than 0.05 was considered statistically significant; ^*^P < 0.05, ^**^P < 0.01, and ^***^P < 0.001 compared with SAHA/bortezomib. Error bars represent the standard error of mean (SEM) of data obtained from the SCID mice (*n* = 5).

We examined the *in vivo* effect of SAHA/bortezomib on growth suppression of EBNA3C-expressing BL xenografts in SCID mice. The mice bearing sLCL 352, 3C-KO BL and 3C-Rev BL xenografts were treated with DMSO (vehicle control, *n* = 6), 50 mg/kg SAHA (*n* = 6), 60 μg/kg bortezomib (*n* = 6), or combination of 50 mg/kg SAHA and 60 μg/kg bortezomib (*n* = 6) for 5 days per week over 18 days (or 24 days for sLCL 352) by IP injection (Figure 7). The growth of tumors was measured during the experiment period (Figure 7A). When compared the two vehicle control groups, tumor volume of 3C-KO (189 mm^3^) and 3C-Rev (236 mm^3^) were similar in Day 1 post-treatment. On day 18, the average tumor volume of 3C-KO xenografts in the groups of mice treated with vehicle control, SAHA, bortezomib and SAHA/bortezomib were 1187 mm^3^, 831 mm^3^, 702 mm^3^ and 675 mm^3^, respectively, whilst those of 3C-Rev xenografts were 2578 mm^3^, 2113 mm^3^, 1991 mm^3^ and 788 mm^3^, respectively. Of note, when compared with SAHA or bortezomib alone, SAHA/bortezomib mediated much stronger growth suppressive effect on 3C-Rev tumors (*p* < 0.01) (Figure 7B). Similar effect of SAHA/bortezomib could also be observed in sLCL 352 xenografts (Figure 7A and 7B). However, such enhanced growth suppression by combination of SAHA/bortezomib was not observed on 3C-KO tumors (Figure 7B). Toxicity of SAHA, bortezomib or their combination in SCID mice was assessed by monitoring their weight (Figure 7C). There was an average of ~5% decrease in weight of mice treated with either single drug or combination of SAHA/bortezomib when compared with the vehicle control group. The data suggested that the specific killing effect of SAHA/bortezomib on EBNA3C-expressing cells could also be observed *in vivo*.

## DISCUSSION

Wp-restricted EBV-positive BL cells and LCLs, which express a broader spectrum of EBV latent proteins, were more resistant to the killing by apoptotic inducers compared with latency I BL cells in which only EBNA1 latent protein was expressed [33, 34]. We reported that Wp-restricted BL cells and LCLs were more resistant to the killing by HDAC inhibitors but the resistance could be overcome by co-administration of a proteasome inhibitor, bortezomib [26]. However, such synergistic killing by SAHA/bortezomib could not be observed in latency I or EBV-negative cell lines [26]. EBNA3 proteins, which are expressed in both Wp-restricted BL and LCLs, could manipulate the cell cycle progression and survival mechanisms in EBV-positive B cells [9, 10, 13]. We had previously shown that tumor suppressor proteins, including p16 and p21^WAF1^, which are down-regulated by EBNA3 protein(s), could be up-regulated by SAHA/bortezomib in a reactive oxygen species (ROS)-dependent manner in Wp-restricted BL cells and LCLs [26]. In this study, we sought to investigate whether SAHA/bortezomib counteracts the survival functions of EBNA3 protein(s) in BL and LCLs.

First, we determined which EBNA3 protein was involved in the synergistic killing by SAHA/bortezomib. We examined the effect of SAHA/bortezomib on the survival of BL cell lines containing EBNA3A/-3B/-3C knockout EBV with or without revertant. We found that SAHA/bortezomib induced significantly greater synergistic killing of 3C-Rev cells when compared with 3C-KO cells. Such differential response was not observed in either EBNA3A- or 3B-Rev versus their KO pairs. These results suggested a more important role of EBNA3C than EBNA3A or EBNA3B in the killing by SAHA/bortezomib. In fact, EBNA3C had been reported to utilize the HDAC enzymes and proteasomeal degradation pathways to facilitate the survival of Wp-restricted BL cells and LCLs [13–15].

We further examined the role of EBNA3C in the cell death induced by SAHA/bortezomib. We found that the cells expressing EBNA3C escaped the G2/M checkpoint arrest induced by SAHA/bortezomib (versus significant G2/M arrest in EBNA3C-knockout cells) and subsequently became more susceptible to the induction of apoptosis by the drug combination. In parallel, SAHA/bortezomib induced stronger expression of p21^WAF1^ but weaker expression of p-cdc25c in EBNA3C-expressing cells when compared with EBNA3C-knockout cells. Moreover, an elevation of cyclin B1 and p-cdc2 proteins level was observed in 3C-KO cells but not in 3C-Rev cells upon treatment with SAHA/bortezomib (data not shown). In line with these observations, EBNA3C was shown to manipulate the cell cycle regulators in the G2/M checkpoints and promote the ubiquitin-proteasome-dependent degradation of p21^WAF1^ in EBV-infected B cells [10, 11, 13, 35, 36]. The fact that bypassing G2/M arrest could lead to enhanced apoptosis was indeed consistently observed in other cell types. For instance, Miyata *et al.* had shown that radiation could induce a stronger apoptosis of esophageal cancer cells after overriding G2/M arrest by overexpression of cdc25b [37]. Wang *et al.* has demonstrated that G2/M arrest induced by a DNA damage agent, curcumin, could protect cancer cells from undergoing apoptosis [29]. Besides, overriding G2/M arrest could result in cell death via mitotic catastrophe in some cancer cell types [38–40]. The exact mechanisms of how the G2/M checkpoint regulation affects cell death may vary among different cell types and require further investigations.

Induction of apoptosis by SAHA/bortezomib involved activation of DNA damage response (DDR) [27, 28]. In response to the DDR, G2/M arrest would be induced through phosphorylation of cdc25c to allow sufficient time for the cells to repair the damaged DNA [29–31, 41–45]. Choudhuri *et al.* had reported that upon treatment with nocodazole, EBNA-3C overrode the DNA damage-induced G2/M arrest by dysregulating cdc25c phosphorylation [11]. Phosphorylation of cdc25c and H2AX was constitutively detected in diffuse large B-cell lymphoma (DLBCL) [46]. Consistently, our data showed that SAHA/bortezomib induced a strong DDR in both BL cells and LCLs as evidenced by the up-regulation of p-H2AX. However, SAHA/bortezomib induced an increased level of cdc25c phosphorylation in 3C-KO cells but not in 3C-Rev or sLCL cells. These data suggested that EBNA3C enabled the EBV-infected BL cells and LCLs to bypass G2/M arrest checkpoint induced by SAHA/bortezomib and rendered the EBV-infected cells more susceptible to the induction of apoptosis. We postulated that the phosphorylation of cdc25c and expression of p21 might possibly be involved in the regulation of the cell death mechanism (refer to Figure 6). However, further detailed investigation of these molecules and other upstream or downstream molecules (e.g. MYC, Bim or p53) is required to define the causal relationships of the molecules involved in this proposed network.

We further evaluated the effect of SAHA/bortezomib on the growth of EBNA3C-positive and EBNA3C-negative B cell xenografts in SCID mice. Our data showed that the *in vitro* anti-tumor effect of SAHA/bortezomib in EBNA3C-expressing BL and spontaneous LCLs could also be achieved *in vivo*. Indeed, previous clinical studies had demonstrated the possible efficacy of SAHA/bortezomib in the treatment of relapsed and refractory multiple myeloma with acceptable toxicities [47, 48]. Some other clinical trials of this drug combination regimen for other disease types (e.g. the refractory or relapsed *Mixed Lineage Leukemia (MLL)*-rearranged hematologic malignancies in young patients) are being actively conducted. In addition to synergistic anti-tumor effect, SAHA/bortezomib could potentially reduce the side effects of either drug alone. For instance, SAHA can reduce bortezomib-induced peripheral neuropathy by inducing autophagy of Schwann cells [49]. The administration of SAHA/bortezomib could also potentially create a room for the reduction of the dosage of chemotherapy for the patients. Our *in vitro* and *in vivo* data provided a strong rationale to test the clinical efficacy of SAHA/bortezomib in the treatment of patients with EBV-associated LPDs which express the EBNA3 proteins, such as PTLD or DLBCL, and particularly in relapsed cases who show resistance to rituximab or chemotherapy.

## MATERIALS AND METHODS

### Cell culture

BL31 parental cell line (BL31 EBV -ve) is an EBV-negative BL cell line. BL31 EBV -WT, BL31 3A-KO, BL31 3A-Rev, BL31 3B-KO, BL31 3B-Rev, BL31 3C-KO, BL31 3C-Rev, BL2 3C-KO and BL2 3C-Rev are all EBV-positive BL cell lines (obtained from Prof. M. Allday, Imperial College, London, UK). All BL cell lines were maintained in RPMI 1640 culture medium supplemented with 10% fetal bovine serum (FBS), 1 mM sodium pyruvate, 50 mM a-thioglycerol and 1% penicillin and streptomycin. 100 mg/ml hygromycin B was used in culture of BL cell lines (except BL31 parental cell line). sLCL 352 and sLCL 381 were established by isolation of EBV-infected peripheral blood mononuclear cells (PBMC) of paediatric PTLD patients and maintained in RPMI 1640 culture medium supplemented with 15% FBS and 1% penicillin and streptomycin. All the cells were grown in a humidified incubator at 37° C with 5% CO_2_.

### Chemicals

SAHA was purchased from Cayman (Cayman Chemicals, Ann Arbor, MI, USA). Bortezomib was purchased from Selleck (Selleck Chemicals, Houston, TX, USA). N-acetyl-cysteine (NAC) was purchased from Sigma (Sigma-Aldrich, St. Louis, MO, USA).

### 3-(4,5-Dimethylthiazol-2-yl)-2,5-diphenyltetrazolium bromide (MTT) assay

BL31, BL2 and sLCLs (5 × 10^5^ cells/ml) were seeded in duplicates in 96-well plates and treated with various concentration of drugs for 24 and/or 48 h. MTT was performed and the percentage of cell proliferation was calculated as previously described [17, 18]. Three independent experiments were performed for each MTT assay.

### Cell cycle analysis

BL31, BL2 and sLCLs (5 × 10^5^ cells/ml) were treated with SAHA and bortezomib for 12, 24, 48 h and 12, 24, 48, 72 h respectively. The treated cells were subjected to cellular DNA content analysis by flow cytometry as previously described [17]. Three independent experiments were performed for each cell line.

### Western blot analysis

BL31, BL2 and sLCLs (5 × 10^5^ cells/ml) were treated with various concentrations of drugs. Protein from the cell cultures was extracted and western blot analysis was performed as previously described [17]. Apoptotic proteins (PARP and Caspese-3) and α-tubulin were detected with the antibodies described previously [18]. EBV latent proteins were detected with rabbit anti-EBNA-1 polyclonal, mouse anti-LMP-1 monoclonal (Prof. Jaap Middeldorp, VU University, Amsterdam, the Netherlands), rat anti-EBNA-2 monoclonal, rat anti-EBNA-3B monoclonal (Prof. Elisabeth Kremmer, Institute of Molecular Immunology, Helmholtz Zentrum München, Munich, Germany), mouse anti-EBNA-LP monoclonal (Prof. Yasushi Kawaguchi, Keio University, Japan), sheep anti-EBNA-3A polyclonal and sheep anti-EBNA-3C polyclonal antibodies (Exalpha Biologiscals, Maynard, MA, USA). Expression of tumor suppressor genes was detected with rabbit anti-p21 monoclonal (Cell Signaling Technology, Beverly, MA, USA) and rabbit anti-p16 polyclonal antibodies (Proteintech, Chicago, IL, USA). Cell cycle related proteins were detected with rabbit anti-CDK4 monoclonal, rabbit anti-cyclin D1 polyclonal, rabbit anti-cyclin B1 monoclonal, rabbit anti-p-cdc2 monoclonal, rabbit anti-p-cdc25C (ser 216) polyclonal (Cell Signaling Technology, Beverly, MA, USA) and rabbit anti-cdc25C (Santa Cruz, California, USA). DNA damage response protein was detected with rabbit anti-p-H2AX (Ser 139) monoclonal (Cell Signaling Technology, Beverly, MA, USA). At least two independent experiments were performed in each western blotting.

### SCID mice experiment

Female C.B-17/Icr-scid (SCID) mice, 5–6 weeks old, were purchased from the Laboratory Animal Unit, the University of Hong Kong. The mice were kept and monitored in LAU under pathogen-free conditions throughout the experiments. All experimental procedures were reported and approved by Committee on the Use of Live Animals in Teaching and Research of the University of Hong Kong. BL31 3CKO (1 × 10^7^), BL31 3CRev (1 × 10^7^) and sLCL 352 (8 × 10^6^) were resuspended in 200 μl of serum-free culture medium (RPMI). Mice, at the age of 6–7 weeks, were subcutaneously injected with one of the above resuspended cells at the right flanks of the mice. When the tumors became palpable, 50 mg/kg SAHA, 60 μg/kg Bortezomib alone or in combination, was dissolved in DMSO in 10 ul and administered to SCID mice of the treatment group (*n* = 6) by intraperitoneal injection (IP) 5 d per week over 18 and 24 days for BL31 and sLCL 352 xenograft-bearing mice respectively. An equal volume of DMSO was administrated by injection to SCID mice of the control group (*n* = 6). The size and weight of the tumors were measured as described previously [18, 23].

### Statistical analysis

All *in vitro* experiments were performed in triplicate and repeated at least 3 times. Data were analyzed for statistical significance using One-way ANOVA Dunnett's Multiple Comparison Test. *P* value < 0.05 was considered statistically significant. Synergism of HDAC and proteasome inhibitors was analyzed with isobologram analysis as described previously [23]. All statistical analyses were performed with GraphPad Prism Version 5.0 software.

## SUPPLEMENTARY MATERIALS FIGURES AND TABLES


