# Supplementary material for: Counteracting survival functions of EBNA3C in Epstein-Barr virus (EBV)-driven lymphoproliferative diseases by combination of SAHA and bortezomib
Source: Oncotarget. 2018 May 18;9(38):25101–14. doi: 10.18632/oncotarget.25341 (PMC5982749; doi:10.18632/oncotarget.25341)
Supplement: Supplementary file 1 [file oncotarget-09-25101-s001.pdf]

Counteracting survival functions of EBNA3C in Epstein-Barr virus (EBV)-driven lymphoproliferative diseases by combination of SAHA and bortezomib

SUPPLEMENTARY MATERIALS

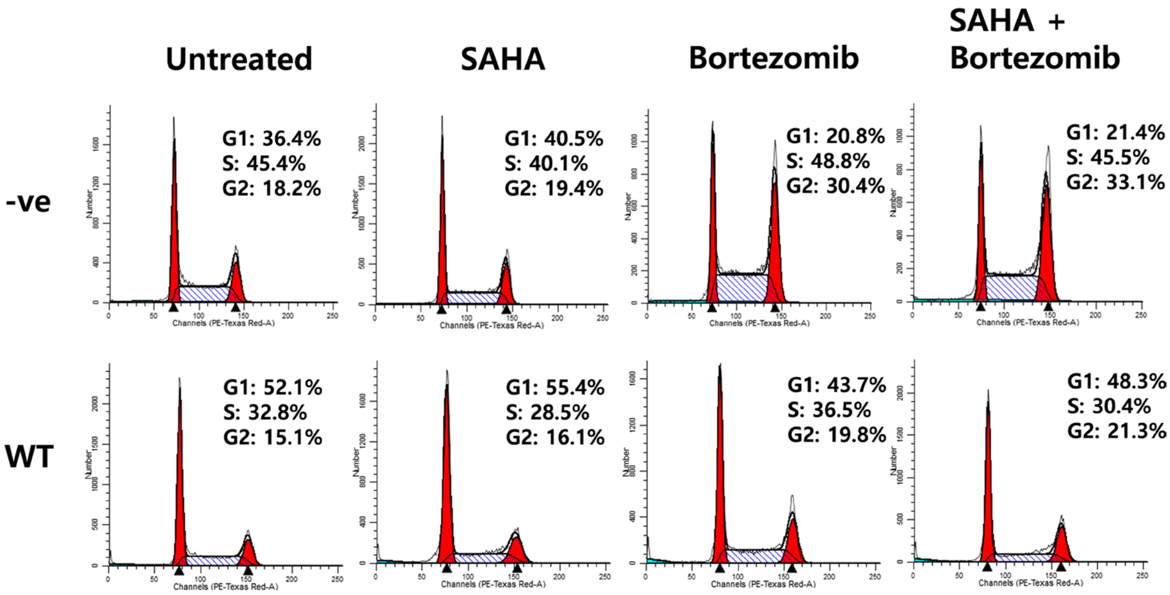

Supplementary Figure 1: Effects of combination of SAHA and bortezomib on cell cycle progression of EBV-negative and EBV-positive BL31 cells. The EBV-negative (EBV-ve) and EBV-positive (WT) BL31 cells were treated with combination of 1 µM SAHA and 8 nM bortezomib or either drugs alone for 12 hr. The treated cells were stained with propidium iodide and subjected to analysis of cellular DNA content by flow cytometry. A representative data set of cell cycle patterns was presented.
